# Analysis of volatile compounds in pork from four different pig breeds using headspace solid‐phase micro‐extraction/gas chromatography–mass spectrometry

**DOI:** 10.1002/fsn3.955

**Published:** 2019-03-27

**Authors:** Guoshun Chen, Yingyu Su, Lianghong He, Hongbin Wu, Shengzhang Shui

**Affiliations:** ^1^ College of Animal Science and Technology Gansu Agricultural University Anning District, Lanzhou China

**Keywords:** flavor, gas chromatography–mass spectrometry, headspace solid‐phase micro‐extraction, pork, volatile compounds

## Abstract

**Purpose:**

The volatile compounds that contribute to the flavor of pork are unknown. Therefore, the present study aimed to determine the differences in volatile compounds from pork meats of four different pig breeds using headspace solid‐phase micro‐extraction (HS‐SPME)/gas chromatography–mass spectrometry (GC‐MS).

**Methods:**

Piglets from four breeds (8/breed) (crossbred Ziwuling *Sus scrofa* [SUS] and purebreds Bamei pig [BAM], American Yorkshire pig [YOK], and Hezuo pig [HZP]) were selected. Characteristics of meat were measured. HS‐SPME/GC‐MS were used to analyze the volatile compounds of the meats.

**Results:**

The tenderness, taste, succulence, and broth flavor of the BAM and HZP were good. One hundred and eight volatile compounds with known molecular formulas were identified in BAM, 106 in SUS, 98 in YOK, and 98 in HZP. Sixty‐four common volatile compounds were found in all four breeds. The highest relative amount of volatile compounds was found in the BAM. The compounds which may contribute to the flavor of pork were 3‐methyl‐1‐butanol, 1‐nonanal, octanal, hexanal, 2‐pentyl‐furan, 1‐penten‐3‐one, N‐morpholinomethyl‐isopropyl‐sulfide, methyl butyrate, and (E,E)‐2, 4‐decadienal.

**Conclusion:**

The volatile compounds in pork belong to several classes, and the highest relative amount of volatile compounds was found in BAM.

## 1 INTRODUCTION

Pork is one of the most widely consumed meat in the world, particularly China with the largest consumption rate (Salter, 2017). With the accelerated restructuring of livestock husbandry, the production and consumption of beef, lamb, and poultry have rapidly increased. However, pork is still predominant in total meat consumption in urban China, accounting for about 70% (Ma, Verkuil, Reinbach, & Meinert, 2017). Pork flavor, an important component of meat taste and aroma, is mainly associated with the generation of volatile compounds (Zhao et al., 2017). Over 1,000 volatile compounds have been identified from cooked meat, including 400 associated with meat flavor (Mottram, 1998). Thus, the volatile compounds associated with the flavor and aroma of meat and meat products should be deeply studied.

Solid‐phase micro‐extraction (SPME) is a technique that allows the efficient detection of compounds and is often used in combination with gas chromatography–mass spectrometry (GC‐MS), high‐performance liquid chromatography (HPLC), and capillary electrophoresis (CE) (Ding, Wu, Huang, & Zhou, 2016; Kataoka, Lord, & Pawliszyn, 2000; Vallarino et al., 2018). Watanabe et al. used a headspace SPME (HS‐SPME) combined with GC‐MS technique to analyze the volatile compounds of different beef samples and revealed significantly higher levels of compounds such as ethyl tetradecanoate and gamma‐nonalactone in Japanese Black cattle than those in beef imported from Australia (Watanabe, Ueda, Higuchi, & Shiba, 2008). Recently, a study showed changing patterns of these different volatile compounds after short‐term starvation, which caused flavor change of fish meat (Jiang, Zhao, Yuan, & Fu, 2017). Genetic and environmental factors have a large impact on the quality of meat of different pig breeds (Pugliese & Sirtori, 2012). However, there is inadequate information on the difference of volatile compounds of different pigs.

Hezuo pig (HZP), also known as Juema pig or Juema wild boar, is a miniature local breed from alpine grazing region. It is a slow‐growing miniature pig with a good amount of lean, thin skin and tenderloin, and agreeable stickiness. Bacon made of it is savory and delicious (Liu & Wang, 2006). Bamei pig (BAM) is good in texture, fresh, tender, and savory, has marbled muscles. Its carcass contains 22.56% proteins, and its ocular muscle has a pH value of 6.71. It is originated from British Yorkshire and its surroundings (Zhou et al., 2016). The American Yorkshire pig (YOK) is white in color, with prick ears. Originally, it is a breed suitable for making bacon, with good texture, but it has become an outstanding lean‐type pig in the United States since the 20th century. YOK is the most widely distributed pig breed in the worldwide. It can be divided into the large, medium, and small types, of which the medium and small types have been reduced or nearly extinct, while the large‐type YOK is all over the world due to strong fertility, thin back fat, rich lean meat, and good texture.

*Sus scrofa*, also known as wild boar or wild pig, is widely distributed worldwide (except Australia, South America, and Antarctica) with 23 recorded subspecies in Asia and six in China (Li & Xu, 1995; Li et al., 2000). A *S. scrofa* breed found in the Ziwuling Mountain at the junction of Shanxi and Gansu Provinces is unique to China. Different purebred and crossbred pigs, raised in various areas, have been used in pork quality research and determination of characteristics of meat flavors, providing an important theoretical basis for the development and utilization of Ziwuling *S. scrofa*, as well as cross‐breeding to improve pork quality. Indeed, the Ziwuling *S. scrofa* breed is known for high intramuscular fat content, superior meat quality, and strong resistance to diseases (Quaresma et al., 2011; Sales & Kotrba, 2013; Yang, Xu, Ma, & Jiang, 2016). Compared with YOK, the Ziwuling *S. scrofa* breed has a low growth rate and fatter meat ratio (Yang et al., 2016). However, purebred wild boar cannot be fed under the same conditions as other pig breeds and it is hard to tame. In addition, stress of raising wild animals may affect the quality of the meat and the number of animals being captured may be a problem. Therefore, the F1 generation of crossbred *S. scrofa* (SUS) was used in this study to determine the differences in volatile compounds of SUS pork, compared with the purebreds BAM, YOK, and HZP using HS‐SPME/GC‐MS.

## 2 MATERIALS AND METHODS

### 2.1 Animals

All animals were fed at a single farm in Zhangye City, Gansu Province, China, in sheds for pig herds with large open spaces, allowing the pigs to freely move around. Sheds were kept dry and clean, with free drinking water and good ventilation. The feeding and management conditions were maintained as consistent as possible. The basic feed was prepared according to the recommendations of the National Research Council (NRC), taking into account the feeding patterns of different pigs. All studies were performed with the approval of the Animal Use and Care Committee of Gansu Agricultural University, China. The feed composition and nutrient levels of basal diets are shown in Table 1.

**Table 1:** Composition and nutrient levels of basal diets

Ingredient	Percent (%)	Nutrient level	Percent (%)
Corn	44.0	Digestible energy (Kcal/kg) DE^a^	3,160
Pop corn	20.0	Crude protein CP	17.5
High‐quality alfalfa meal	6.0	Ether extract EE	3.50
Soybean meal	14.0	Dry matter DM	82.6
Wheat bran	3.0	Ash	4.98
Fermented soybean meal	6.0	Ca	0.85
Domestic fish meal	3.0	Total phosphorus TP	0.63
4% Premix^b^	4.0	Salt	0.45

*Note*

^a^Calculated values. ^b^Nutrients in the premix contained (per kg of feed) the following: Fe, 85.0 mg; Zn, 82.0 mg; Mn, 44.0 mg; Cu, 29.0 mg; Se, 0.35 mg; I, 0.68 mg; vitamin A, 810 IU; vitamin D_3_, 950 IU; vitamin E, 48.0 mg; vitamin B_1_, 2.15 mg; vitamin B_2_, 2.80 mg; biotin, 0.06 mg; folic acid, 0.34 mg; niacin, 30.00 mg; calcium pantothenate, 26.00 mg; vitamin B_6_, 1.00 mg; vitamin B_12_, 0.01 mg; choline chloride, 450.0 mg; antioxidants, 25.0 mg.

A Ziwuling *S. scrofa* male was captured, kept in a local Guihuayuan pig farm, and crossbred with a female of the Gansu local breed Bamei pig, to produce the F1 generation of SUS. In this study, four breeds of animals were evaluated: SUS (F1 generation crossbred), BAM, YOK, and HZP. In each group, the same paternal line was mated with 2–3 maternal lines of the same breed to obtain piglets. Then, eight weaned piglets were selected from the 2–3 maternal lines of each group. All selected animals were in the same growth conditions, and the weight difference was <0.5 kg (Table 2). These pigs were bred for 100 days under the same conditions for determining production performances, and then were slaughtered for determination of meat texture. All visible fat was removed during sample preparation. Before analyzing for volatile components, the samples were tested for microbiological populations.

**Table 2:** Characteristics of the meats of four different pig breeds

	SUS	BAM	YOK	HZP
*n*	8	8	8	8
Carcass quality				
Percentage of dressed weight	68.83 ± 1.83^a^	74.00 ± 1.23^b^	75.63 ± 1.89^b^	66.13 ± 2.41^a^
Carcass length (cm)	60.60 ± 3.36^b^	80.05 ± 4.76^a^	82.12 ± 3.11^a^	47.50 ± 2.12^b^
Back fat thickness (cm)	2.11 ± 0.22	3.13 ± 0.43	2.78 ± 1.12	2.50 ± 0.33
Skin thickness (cm)	0.368 ± 0.03^a^	0.301 ± 0.02	0.240 ± 0.06^b^	0.220 ± 0.01^b^
Eye muscle area (cm^2^)	29.69 ± 4.78^b^	39.78 ± 4.96	47.34 ± 7.62^a^	18.64 ± 3.04^b^
Ratio of hindquarter (%)	30.0 ± 2.46	30.8 ± 3.38	29.7 ± 5.95	31.56 ± 3.75
Meat qualities				
pH_1_	5.74 ± 0.33	6.13 ± 0.03	6.23 ± 0.17	6.45 ± 0.12
Percentage of water loss	33.07 ± 1.58^a^	32.39 ± 1.04^a^	34.01 ± 5.54^a^	10.68 ± 0.73^b^
Meat color				
*L*	35.36 ± 2.34	35.17 ± 2.89	34.96 ± 2.58	36.18 ± 3.12
*a*	6.54 ± 0.578^ab^	6.29 ± 0.688^ab^	5.12 ± 0.55^b^	7.61 ± 0.678^a^
*b*	7.74 ± 0.698^a^	7.13 ± 0.638^a^	4.84 ± 0.51^b^	7.53 ± 0.718^a^
Marbling (points)	2.75 ± 0.35^b^	4.00 ± 0.14^a^	2.77 ± 0.29^b^	3.92 ± 0.04^a^
Cooking rate (%)	66.78 ± 3.58	70.00 ± 4.71	67.98 ± 1.03	70.37 ± 0.87
Storage loss (%)	2.09 ± 0.26^c^	2.78 ± 0.08^b^	3.35 ± 0.22^a^	1.84 ± 0.12^c^
Percentage of lean meat	62.27	55.64	63.26	56.56
Percentage of dressing	70.83	74	75.63	66.13
Taste identification				
Tenderness	8.5 ± 0.15	8.6 ± 0.12	8.4 ± 0.12	8.7 ± 0.14
Taste	8.8 ± 0.13	8.7 ± 0.11	8.2 ± 0.14	8.8 ± 0.11
Succulence	8.4 ± 0.11	9.3 ± 0.10	8.2 ± 0.13	9.5 ± 0.09
Broth flavor	8.6 ± 0.14	8.5 ± 0.12	8.3 ± 0.16	8.6 ± 0.12
Fiber diameter (μm)	48.55 ± 6.83	40.14 ± 9.48	42.79 ± 1.76	37.04 ± 1.42
Moisture	69.18 ± 1.87	72.38 ± 0.48	70.10 ± 9.10	74.34 ± 5.46

*Note*

Data are shown as mean ± *SEM*. Values with same superscript letters or no superscript letters in the same line do not differ significantly (*p *>* *0.05). Values with superscript letters in the same line were significantly different (*p *<* *0.05). Values with superscript letters between a and c in the same line were highly significantly different (*p *<* *0.01).

### 2.2 Taste identification

Meat samples were boiled without sauce, sliced, and placed into a dinner plate. Then, 10 experts in animal by‐products assessed tenderness, taste, succulence, and broth flavor. Taste was evaluated using a 10‐point method: scores >8.5, 8.5–7.0, and 7.0–6.0 referred to good, intermediate, and poor taste, respectively.

### 2.3 Meat color

The CR‐400 type color difference meter (Hangzhou Ke Sheng Instrument Co., Ltd.) was adopted in the experiment. The flesh color in the eye muscle was determined after slaughter for 45 min, and the difference was judged according to the measured value of *L*,* a*, and *b*.

### 2.4 PH_1_ value

PH_1_ value was determined within 45 min after slaughter and was directly performed by puncturing the longissimus dorsi muscle at the last but two and three thoracic vertebra. The procedures were in accordance with the instructions of pH meter (pH210) or digital pH meter (DHS‐2F). Holes were punctured in the meat sample using a knife. Then, the electrode was directly inserted into the central puncture hole, at a depth to ensure that the electrode head was completely embedded in the meat sample (1.0–2.0 cm). Then, the pH_1_ value was read (precision of 0.01). Normal pH_1_ value was 6.0–6.6. If the meat had a pH_1_ <5.9, accompanied by gray color and a large amount of exudative fluids, it was judged as a PSE meat. For hybrid swine, the lower limit of normal pH_1_ was 5.6 according to the slaughtering circumstances and referring to individual stress‐sensitive breeds (such as Pietrain). It is because that the hybrid swine is impatient, shy, and difficult to catch, which results in a long slaughtering duration, and is likely to cause effects on meat quality and result in a low acidity.

### 2.5 Water loss percentage

Percentage of water loss was determined using the pressure method at room temperature. The longissimus dorsi muscle at the last and the last but one thoracic vertebra was collected within 45 min after the pigs were slaughtered. Circular meat samples (area of 5 cm^2^ and thickness of 1 cm) were cut using a circular cutter with a diameter of 2.532 cm, and were weighted. Then, the circular meat sample was sandwiched between two layers of gauze and 18 layers of qualitative filter paper were applied to both sides. The samples were pressurized to 35 kg (stress of 138.8 kPa) for 5 min. After the pressure was removed, meat sample was stripped from the gauze and weighted. The percentage of water loss and water holding capacity was given by: Water loss percentage (%) = [(pre‐pressure weight−post‐pressure weight)/pre‐pressured weight] × 100%.

### 2.6 Marbling

Marbling was evaluated using US‐made NCCP colorimetric plate (1991 edition). Fresh meat samples were cut from the longissimus dorsi muscle at the thoracolumbar junction (thickness not <1.5 cm). Marbling was evaluated at the same time as meat color using the same testing conditions. The meat sample was scored by comparison with the colorimetric plate: 1, 2, 3, 4, and 5 points referred to trace amount, micro amount, moderate amount, plenty amount, and excessive amount of fat.

### 2.7 Cooked meat percentage

The greater psoas muscle was collected from the left carcass, from which about 500 g (W_1_) of meat was cut off, weighted, and labeled. Then, the meat sample was placed in an aluminum cooker, added with an appropriate amount of cold water, and steamed on a 2,000 electric stove for 45 min after the water started to boil. Subsequently, the meat sample was taken out and hung for 30 min, followed by weighting (W_2_). Cooked meat percentage (%) = W_2_/W_1_ × 100%.

### 2.8 Area of eye muscle

Parchment paper was firmly attached to the cross section of the thoracolumbar junction of the hot carcass (left side), and the profile of the cross section was depicted using a panicle, which was brought back to the laboratory for measurement using a planometer. Area of the eye muscle (cm^2^) = Length × width × 0.7.

### 2.9 Meat tenderness

Loin‐eye muscle was collected within 45 min after the pigs were slaughtered, then immersed into a water bath at 75–80°C until the core temperature reached 70°C. Then, the meat sample was taken out and cooled to room temperature. Meat piece with a width of 1.5 cm was cut perpendicular to the muscle fibers. Meat pieces were cut along the muscle fiber direction using a circular cutter (diameter of 1.27 cm). Ten samples were obtained from each animal. Meat tenderness was measured using a C‐LM3 digital meat tenderness instrument developed by the Engineering College of the Northeast Agricultural University. The shear forces of the 10 meat pieces were recorded, and their average value was used for analysis as N or kg.

### 2.10 Chemical composition of meat

The 105°C constant weight method was selected to determine the moisture according to the standard GB 5009.3‐85. Crude protein content was determined using the Kjeldahl method (semi‐micro steam distillation) according to standard GB 5009.5‐85. The Soxtec (HT) system (Tecator Digestion Systems, Eden Prairie, MN, USA) was used to analyze the crude fat content. Ash content was determined using the ignition loss method according to standard GB5009.4‐85. For amino acid content, the meat sample was dried, degreased, and hydrolyzed with 6 N HCL. Then, the contents of several amino acids were determined using the WATERS 600 HPLC System (Waters, Milford, MA, USA). The GC‐14CPTF gas chromatography system was used to determine the contents of palmitic acid, linoleic acid, oleic acid, linolenic acid, and other important fatty acids. Atomic absorption spectroscopy was used to determine the calcium and phosphorous contents of the samples according to standard GB 12398‐90 and GB 12393‐90.

### 2.11 Sample preparation and extraction procedure

The longissimus muscles at the last and second last ribs were extracted. The meat samples (120 g) from each piglet breeds were grinded, divided into four parts, and placed in sealed glass bottles. One part was randomly selected for homogenization; the remaining parts were kept at −27°C. The sealed vials were incubated at room temperature for 40 min and then placed in an incubator at 80°C for 40 min for activation (Wang, Wang, Liu, & Chen, 2008). Then, 6 g of activated samples was placed into headspace vials. The syringe plunger was pushed to head out the fiber from the needle, and the fiber was placed into the top space (headspace mode) for extraction for 40 min and then subsequently at room temperature using the SPME technique. The fiber head was retracted, and the needle was withdrawn from the vial. The SPME needle was inserted into the GC‐MS inlet, and the syringe plunger was pushed to expose the fiber for thermal desorption, followed by column chromatography analysis during which a manual SPME injector was used. The fiber was retracted, and the needle was removed. For each sample, 100 μm of polydimethylsiloxane (PDMS) fiber and 85 μm of polyacrylate fiber (Supelco, Sigma, St Louis, MI, USA) were used and pooled.

### 2.12 Gas chromatography–mass spectrometry

Gas chromatography–mass spectrometry was carried out on a Finnigan TRACE MS (Thermo Fisher Scientific, Waltham, MA, USA) equipped with a PEG 20M capillary column (30 m long, 0.25 mm diameter, 0.25 μm film thickness, Beijing Keyi Hengda Technology Co., Beijing, China). Helium was used as carrier gas in the splitless mode at constant flow of 0.8 ml/min. The inlet and interface temperature was 250°C, and for separation, an initial column temperature of 35°C for 5 min was followed by a gradual increase of 5°C/min to 230°C, held for 8 min. MS conditions were as follows: ion source temperature, 200°C; ionization, electron impact (EI); electron energy, 70ev; mass scanning range: 33–500 atomic mass units (amu).

Identification of compounds was initially carried out by searching the NIST02 spectrum library and referring to literature about the volatile compounds in pork, followed by confirmation using CI‐derived (M + 1) quasi‐molecular ions. Quantification was carried out by normalizing the area of an ion to the total ion chromatogram.

### 2.13 Statistical analysis

All statistical analyses were performed using SPSS 13.0 (SPSS Inc., Chicago, IL, USA). Data were expressed as mean ± standard error of mean (*SEM*). Statistical significance was evaluated by one‐way analysis of variance (ANOVA) with the Duncan test for post hoc analysis. The *p*‐value <0.05 was considered statistically significant.

## 3 RESULTS

### 3.1 Characteristics of meat

As can be seen from Tables 2 and 3, the carcass length was longer in the BAM and YOK groups compared with the SUS and HZP groups (*p *<* *0.05). The skin was thicker in the SUS group compared with the YOK and HZP groups (*p *<* *0.05). Eye muscle area was larger in the YOK group compared with the SUS and HZP groups (*p *<* *0.05). The percentage of water loss was smaller in the HZP group compared with the other groups (*p *<* *0.05). The *L* values of flesh color were not significantly different among the four groups (*p* > 0.05). The meat color *a* and *b* values in the HZP group were significantly higher than those of the YOK group (*p* < 0.05), but there was no significant difference between the meat color *a* value and *b* value of SUS and BAM (*p* > 0.05). Meat color and marbling were better in the HZP group (*p *<* *0.05). The HZP and SUS had the smaller percentage of storage loss (*p *<* *0.05).

**Table 3:** Chemical analysis of the meats of four different pig breeds

	SUS	BAM	YOK	HZP
*n*	8	8	8	8
Composition of the meat (%)				
Crude protein	24.47 ± 1.01^a^	22.10 ± 0.04^b^	22.28 ± 1.17^b^	20.78 ± 3.96^b^
Intramuscular fat	3.51 ± 0.28^b^	3.01 ± 0.34^b^	6.34 ± 2.43^a^	3.46 ± 2.15^b^
Ash	1.21 ± 0.40	1.20 ± 0.11	0.95 ± 0.07	1.11 ± 0.24
Ca	0.16 ± 0.02	0.05 ± 0.01	0.05 ± 0.01	0.06 ± 0.01
P	0.33 ± 0.00	0.21 ± 0.01	0.23 ± 0.01	0.24 ± 0.01
Amino acid composition in muscle (mg/100 mg) of dried meat (%)				
Aspartic acid	9.26	7.19	6.83	7.60
Glycine	4.82	5.00	4.85	4.54
Glutamic acid	11.51	8.44	7.33	10.51
Alanine	2.26	1.89	1.74	1.26
Proline	3.63	2.98	1.73	2.92
Total AA	70.10	77.61	73.39	68.03
Total EAA	31.01	33.07	30.81	30.09
EAA/total AA	39.96	45.06	45.29	42.92
Flavors AA	27.85	22.52	20.75	23.91
Flavors AA/total AA	35.88	30.69	30.50	34.50
Fatty acid composition (%)				
Oleic acid (C18:1)	38.15	35.86	37.02	39.84
Linoleic acid (18:2)	5.22	7.31	4.30	7.61
Linolenic acid (18:3)	6.05	4.61	3.97	6.54
Other fatty acid	6.17	0.62	3.28	4.48
SFA	44.41	51.6	51.43	41.53
UFA	49.42	47.78	45.29	53.99
UFA/SFA	1.11	0.93	0.88	1.30

*Notes*

Data are shown as mean ± *SEM*. Values with same superscript letters or no superscript letters in the same line do not differ significantly (*p *>* *0.05). Values with superscript letters in the same line were significantly different (*p *<* *0.05). Values with superscript letters between a and c in the same line were highly significantly different (*p *<* *0.01).

AA: amino acids; EAA: essential amino acids; SFA: saturated fatty acids; UFA: unsaturated fatty acids.

The tenderness, taste, succulence, and broth flavor of the BAM and HZP were good. In the SUS, except that succulence was moderate, the other indicators were good. Meanwhile, all the indicators of the YOK pigs were moderate. There were no differences among the four groups for these characteristics.

Crude protein content was higher in the SUS group compared with the other groups (all *p *<* *0.05), while intramuscular fat was higher in the YOK (all *p *<* *0.05). All other parameters were similar among the four groups.

### 3.2 Comparison of volatile compounds in pork meats from all breeds

Over 100 peaks were found in each gas chromatogram (Supporting Information Figure S1). Among them, 108 volatile compounds with known molecular formulas were identified in BAM, the largest number among all breeds, followed by 106 compounds in SUS. In YOK and HZP, only 98 compounds were identified from pork meats. The relative amounts of total volatile compounds with known molecular formulas accounted for 73.020%, 99.957%, 75.877%, and 76.996% of all detected compounds in SUS, BAM, YOK, and HZP, respectively. The highest relative amount of the unidentified compounds was found in the crossbred SUS, followed by YOK and HZP, while the lowest was found in BAM.

Sixty‐four common volatile compounds were observed in the various pork meats studied, at different amounts (Table 4). BAM showed the highest sum of relative amount (67.991%) of common volatile compounds, followed by HZP (58.754%), YOK (49.172%), and SUS (42.478%). Trimethyl fluorosilane was the most abundant volatile compound in all breeds. Compounds that showed marked differences in their relative amounts included allyl isobutyrate, (Z)‐2‐penten‐1‐ol, 3‐ethyl‐2,2‐dimethyl‐oxirane, fluorotrimethylsilane, 2‐pentyl‐furan, D‐Lilac alcohol, octanal, dodecane, N‐decanoic acid, acetate‐1H‐indole‐3‐ethanol, eicosane, 3‐methyl‐1‐butanol, 2‐decene‐1‐ol, and (1‐demethyl)‐benzene (all *p *<* *0.05). For example, the relative amounts of 2‐pentyl‐furan were significantly higher in SUS than in BAM, YOK, and HZP (all *p *<* *0.05), while no significant differences were observed among the BAM, YOK, and HZP (all *p *>* *0.05) groups. Interestingly, the relative amounts of D‐lilac alcohol, octanal, dodecane, N‐decanoic acid, eicosane, 3‐methyl‐1‐butanol, 2‐decene‐1‐ol, and (1‐demethyl)‐benzene were markedly different among the groups (*p *<* *0.01). The detailed comparison of all volatile compounds is shown in Table 4.

**Table 4:** Comparison of the same flavor compound within different pigs and their relative content (%)

	Compound name	SUS	BAM	YOK	HZP
1	Allyl ester isobutyric acid	0.809	3.447	1.324	3.265
2	(Z)‐2‐ 5 methylamine ‐1‐ alcohol	2.397	4.216	2.204	3.936
3	3‐methyl‐2, 2‐dimethyl ethylene oxide	0.390	1.064	1.479	0.993
4	2‐butyl‐3‐methyl‐ethylene oxide	0.056	0.683	0.534	0.59
5	Fluorotrimethylsilane	12.942	13.593	16.146	14.211
6	2, 4‐Dimethylheptane	0.341	0.267	0.208	0.356
7	1,3, 5‐tricyclohexyl hexahydride‐1,3, 5‐ triazine	0.742	0.77	0.893	0.721
8	2‐Pentyl‐furan	3.174	2.326	1.833	2.172
9	2‐Undecane	1.131	1.183	1.077	1.105
10	Heptyl aldehyde	0.226	0.078	0.05	0.073
11	3‐Bromo‐pentene	0.133	0.216	0.190	0.202
12	D‐butyl sweet	2.950	4.292	3.508	4.007
13	1‐Heptanol	0.759	1.332	1.136	1.246
14	Anti‐4‐cyclopentene‐1, 3‐diol	0.076	0.038	0.043	0.036
15	Caprylic aldehyde	0.377	0.227	0.259	1.012
16	1, 2‐Dimethyl‐3‐cyclopentyl alcohol	0.082	0.09	0.067	0.108
17	Undecane	0.116	0.188	0.126	0.175
18	1‐Chloro‐octane	0.417	0.547	0.481	0.511
19	4‐Ethyl cyclohexanol	0.079	0.201	0.159	0.187
20	1‐Pentene‐3‐ketone	0.107	0.134	0.105	0.125
21	1‐Octanol	0.359	0.648	0.565	0.795
22	5, 5‐Dimethyl‐1, 3‐heptadiene	0.56	0.349	0.307	0.326
23	(Z)2‐octene‐1‐ol	0.533	0.916	0.844	0.855
24	(E)‐2‐caprylic aldehyde	0.792	1.905	1.204	1.821
25	Ethyl 2‐propylene‐2‐butyrate	0.413	0.411	0.846	0.385
26	1‐Nonyl aldehyde	0.368	0.514	0.361	0.580
27	Dodecane	0.085	0.090	0.544	0.084
28	Butyl‐ethylene oxide	0.015	0.071	0.021	0.066
29	2‐bromo‐6‐methylheptane	0.020	0.087	0.015	0.105
30	1‐decyl alcohol	0.21	0.312	0.418	0.292
31	Cis‐4‐decanal	0.064	0.106	0.08	1.199
32	Tridecane	0.052	0.174	0.07	0.162
33	Hexanal	0.173	0.276	0.181	0.364
34	Pelargonic acid	0.104	0.141	0.072	0.131
35	Pentadecane	0.021	0.078	0.028	0.073
36	(E,E)‐2,4‐decylene aldehyde	0.066	0.126	0.103	0.318
37	1‐Tetradecene	0.104	0.141	0.072	0.131
38	Styrene acrylic thiazole	0.194	0.548	0.674	0.512
39	2,4‐Decylene aldehyde	0.351	0.439	0.092	0.410
40	4‐methyl‐2‐hydroxy‐cyclopentene‐2‐ene‐ 1‐ketone	0.117	0.043	0.074	0.040
41	N‐decanoic acid	0.076	0.29	0.122	0.271
42	5‐ Methyl‐ethyl ester‐nonanoic acid	0.024	0.052	0.021	0.048
43	NONA‐3,5‐ diethylenetriamine‐2‐ ketone	0.149	0.159	0.066	0.15
44	Hexadecyl aldehyde	0.024	0.071	0.036	0.071
45	Tetradecyl	0.035	0.105	0.044	0.098
46	4‐(1‐methylethyl)‐2‐cyclohexene‐1‐ketone	0.047	0.068	0.043	0.063
47	7‐Methyl‐3‐octene‐2‐ketone	0.04	0.09	0.067	0.084
48	2, 9‐Dimethyl‐decane	0.113	0.178	0.049	0.166
49	2‐(1‐methylethyl)‐1, 3‐dioxopentane	0.053	0.063	0.093	0.059
50	Pentadecane	0.217	0.483	0.34	0.451
51	2, 6‐Dimethyl‐undecane	0.211	0.209	0.413	0.195
52	Acetic ester‐1H‐indole‐3‐ethanol	0.120	0.394	0.110	0.368
53	17 (alkyl) aldehyde	0.055	0.096	0.088	0.089
54	Diethyl phthalate	0.047	0.061	0.021	0.057
55	1‐Bromo‐2‐methyl‐decane	0.028	0.091	0.081	0.108
56	Vinyl silane	2.534	3.824	2.762	3.570
57	Eicosane	1.614	0.164	0.1304	0.153
58	Four hydrogen‐3‐methyl‐2‐beta furan formic acid	0.052	0.147	0.063	0.137
59	3‐Methyl‐1‐butanol	1.249	0.611	0.278	0.537
60	N‐Hexadecanoic acid	0.128	0.103	0.1	0.096
61	2,3, 5‐Trimethylhexane	0.148	0.112	0.102	0.104
62	2‐Decene‐1‐ol	0.403	10.69	0.222	0.998
63	(1‐ to methyl)‐benzene	0.809	3.447	1.324	3.265
64	Double (2‐methacrylate)‐1, 2‐phthalic acid	2.397	4.216	4.204	3.936
	The same compound as the percentage of compounds	42.478	67.991	49.172	58.754

### 3.3 The relative amounts of other volatile compounds from specific breeds

In total, 34–44 other different volatile compounds were found in the four pork breeds (Table 5). The relative amounts of these compounds were different, and their sum in SUS, BAM, YOK, and HZP accounted for 30.542%, 31.966%, 26.705%, and 25.553%, respectively. The highest relative amounts were found in BAM, and the lowest in HZP. 3,5‐Dimethylfuran was exclusively detected in BAM samples but not in the meats of other breeds.

**Table 5:** Comparison of the contents of other identified volatile compounds within different pig breeds

Number	SUS				BAM				YOK				HZP			
	Name	Formula	Molecular weight	Amount (%)	Name	Formula	Molecular weight	Amount (%)	Name	Formula	Molecular weight	Amount (%)	Name	Formula	Molecular weight	Amount (%)
1	Isopentane	C_5_H_12_	72.15	4.933 ± 1.220	Acetic acid	C_2_H_4_O_2_	60.05	3.154 ± 0.780	Acetic acid	C_2_H_4_O_2_	60.05	3.195 ± 0.790	1‐Octane	C_8_H_18_	114.23	1.477 ± 0.246
2	1‐Octene‐3‐ol	C_8_H_16_O	128.21	0.320 ± 0.056	1‐Octane	C_8_H_18_	114.23	1.561 ± 0.274	1‐Octane	C_8_H_18_	114.23	1.582 ± 0.278	1‐Octene‐3‐ol	C_8_H_16_O	128.21	1.897 ± 0.280
3	Chloroisopentane	C_5_H_11_Cl	106.59	0.557 ± 0.137	Ethyl benzene	C_8_H_10_	106.17	1.012 ± 0.249	1‐Octene‐3‐ol	C_8_H_16_O	128.21	1.869 ± 0.460	Ethyl benzene	C_8_H_10_	106.17	0.958 ± 0.237
4	(Z)‐2‐Octane	C_8_H_16_	112.21	0.242 ± 0.048	(Z)‐2‐Octene‐1‐ol	C_8_H_16_O	128.21	0.024 ± 0.005	Ethyl benzene	C_8_H_10_	106.17	1.026 ± 0.203	(Z)‐2‐Heptenal	C_7_H_12_O	112.17	0.914 ± 0.209
5	1,2,4‐Trimethyl cyclohexane	C_9_H_18_	126.24	0.209 ± 0.052	(Z)‐ 2‐Heptenal	C_7_H_12_O	112.17	0.966 ± 0.239	(Z)‐2‐Octene‐1‐ol	C_8_H_16_O	128.21	0.024 ± 0.006	2‐Undecanal	C_11_H_20_O	168.28	0.140 ± 0.029
6	1‐Hexanol	C_6_H_14_O	102.17	0.423 ± 0.084	2‐Undecanal	C_11_H_20_O	168.28	0.148 ± 0.029	2‐Heptenal,(2Z)	C_7_H_12_O	112.17	0.979 ± 0.194	2‐Ketopalmitic acid methyl ester	C_17_H_32_O_3_	284.43	0.080 ± 0.018
7	(Z)‐2‐Octene‐1‐ol	C_8_H_16_O	128.21	0.097 ± 0.019	2‐Ketopalmitic acid methyl ester	C_17_H_32_O_3_	284.43	0.085 ± 0.017	2‐Undecanal	C_11_H_20_O	168.28	0.15 ± 0.030	Undecanoic acid	C_11_H_22_O_2_	186.29	0.247 ± 0.051
8	2,3,5‐Trimethyl cyclohexane	C_9_H_20_	128.26	0.149 ± 0.026	Undecanoic acid	C_11_H_22_O_2_	186.29	0.261 ± 0.046	2‐Ketopalmitic acid methyl ester	C_17_H_32_O_3_	284.43	0.066 ± 0.012	Dodecane,2,7,10‐trimethyl‐	C_15_H_32_	212.41	0.062 ± 0.014
9	Dodecyl aldehyde	C_12_H_24_O	184.32	0.058 ± 0.014	Dodecane,2,7,10‐trimethyl‐	C_15_H_32_	212.41	0.065 ± 0.016	Undecanoic acid	C_11_H_22_O_2_	186.29	0.264 ± 0.065	Phenol,4‐(2‐aminopropyl)‐	C_9_H_13_NO	151.20	1.445 ± 0.270
10	2,6‐Dimethylheptadecane	C_19_H_40_	268.52	0.177 ± 0.033	4‐(2‐aminopropyl)‐ Phenol,	C_9_H_13_NO	151.20	1.528 ± 0.286	Dodecane,2,7,10‐trimethyl‐	C_15_H_32_	212.41	0.066 ± 0.012	2,2′‐(1,2‐ethenediyl)bis[5‐isothiocyanato‐ Benzenesulfonic acid	C_16_H_10_N_2_O_6_S_4_	454.52	7.045 ± 1.319
11	Dodecane,2,7,10‐trimethyl‐	C_15_H_32_	212.41	0.047 ± 0.009	3‐methyl‐Pentanal	C_6_H_12_O	100.16	2.143 ± 0.425	4‐(2‐aminopropyl)‐ Phenol	C_9_H_13_NO	151.20	1.548 ± 0.290	3‐methyl‐Pentanal,	C_6_H_12_O	100.16	2.027 ± 0.465
12	Diisobutyl phthalate 1,2‐Benzenedicarboxylic acid bis(2‐methylpropyl)ester	C_16_H_22_O	278.34	0.649 ± 0.150	2,2′‐(1,2‐ethenediyl)bis[5‐isothiocyanato‐Benzenesulfoc acid	C_16_H_10_N_2_O_6_S_4_	454.52	7.445 ± 1.394	2,2′‐(1,2‐ethenediyl)bis[5‐isothiocyanato‐Benzenesulfonic acid	C_16_H_10_N_2_O_6_S4	454.52	7.037 ± 1.624	3‐Benzoyl‐4‐phenyl‐5,6‐dihydrothiopyran	C_18_H_16_OS	280.43	0.953 ± 0.195
13	1‐Decene,3,4‐dimethyl‐	C_12_H_24_	168.32	0.197 ± 0.039	3‐Benzoyl‐4‐phenyl‐5,6‐dihydro thiopyran	C_18_H_16_OS	280.43	1.104 ± 0.201	3‐Benzoyl‐4‐phenyl‐5,6‐dihydro thiopyran	C_18_H_16_OS	280.43	1.021 ± 0.202	Acetyl acetaldehyde Acetamido acetaldehyde	C_4_H_7_O_2_N	101.23	0.973 ± 0.182
14	(Z)‐2‐Heptenal 2‐Heptenal,(2Z)‐	C_7_H_12_O	112.16	0.463 ± 0.092	Acetyl acetaldehyde	C_4_H_7_O_2_N	101.23	1.028 ± 0.204	Pentanal,3‐methyl‐	C_6_H_12_O	100.16	2.171 ± 0.431	2‐Octanone	C_8_H_16_O	128.21	0.210 ± 0.044
15	(E,E)‐2,4‐Dodecadienal	C_12_H_20_O	180.29	0.041 ± 0.008	Gamma‐Butyrolactone	C_4_H_6_O_2_	86.09	0.177 ± 0.035	Acetyl acetaldehyde	C_4_H_7_O_2_N	101.23	1.042 ± 0.203	Gamma‐Butyrolactone	C_4_H_6_O_2_	86.09	0.163 ± 0.027
16	2‐Undecenal	C_11_H_20_O	168.28	0.069 ± 0.014	Propanoicacid, ethenylester	C_5_H_8_O_2_	100.11	0.13 ± 0.026	2‐Octanone	C_8_H_16_O	128.21	0.225 ± 0.045	Propanoicacid,ethenylester	C_5_H_8_O_2_	100.11	0.123 ± 0.026
17	2‐Ketopalmitic acid methyl ester	C_17_H_32_O3	284.43	0.035 ± 0.007	(E,E)‐3,5‐octadiene‐2‐one (E,E)‐3,5‐Octadiene‐2‐one	C_8_H_12_O	124.18	0.276 ± 0.052	Propanoicacid,ethenylester	C_5_H_8_O_2_	100.11	0.132 ± 0.025	(E,E)‐3,5‐octadiene‐2‐one (E,E)‐3,5‐Octadiene‐2‐one	C_8_H_12_O	124.18	0.262 ± 0.046
18	N‐ Morpholinomethyl ‐isopropyl‐sulfide	C_8_H_17_ONS	175.29	0.438 ± 0.087	ethane,1‐(3‐butenyloxy)‐ Hexane,1‐(3‐butenyloxy)‐	C_10_H_20_O	156.27	0.411 ± 0.077	(E,E)‐3,5‐octadiene‐2‐one (E,E)‐3,5‐Octadiene‐2‐one	C_8_H_12_O	124.18	0.275 ± 0.055	ethane,1‐(3‐butenyloxy)‐ Hexane,1‐(3‐butenyloxy)‐	C_10_H_20_O	156.27	0.389 ± 0.096
19	Undecanoic acid	C_11_H_22_O_2_	186.29	0.142 ± 0.027	Cyclohexane,(3‐methylpentyl)‐	C_12_H_24_	168.32	0.066 ± 0.013	ethane,1‐(3‐butenyloxy)‐ Hexane,1‐(3‐butenyloxy)‐	C_10_H_20_O	156.27	0.417 ± 0.078	Cyclohexane,(3‐methylpentyl)‐	C_12_H_24_	168.32	0.063 ± 0.014
20	Dodecane,2,7,10‐trimethyl‐	C_15_H_32_	212.41	0.050 ± 0.009	6,10‐Dimethylundeca‐5,9‐dien‐2‐one	C_13_H_22_O	194.31	0.021 ± 0.004	Cyclohexane,(3‐methylpentyl)‐	C_12_H_24_	168.32	0.067 ± 0.013	6,10‐Dimethyl‐5,9‐undecadien‐2‐one Geranylacetone	C_13_H_22_O	194.31	0.020 ± 0.004
21	Ethyl benzene	C_8_H_10_	106.17	0.040 ± 0.008	1‐ Nonene ‐3‐ alcohol	C_9_H_18_O	142.24	0.009 ± 0.002	6,10‐Dimethyl‐5,9‐undecadien‐2‐one Geranylacetone	C_13_H_22_O	194.31	0.022 ± 0.007	Malonicacid,bis(2‐trimethylailylethyl)ethyl ester	C_13_H_28_O_4_Si_2_	304.73	0.302 ± 0.049
22	11‐Octadecenoic acid,(11E)‐	C_18_H_34_O_2_	282.46	0.046 ± 0.009	Heptadecane	C_17_H_36_	240.47	0.211 ± 0.070	Heptadecane	C_17_H_36_	240.47	0.214 ± 0.042	Heptadecane	C_17_H_36_	240.47	0.200 ± 0.031
23	Acetic acid	C_2_H_4_O_2_	60.05	3.065 ± 0.608	Malonicacid,bis(2‐trimethylailylethyl)ethyl ester	C_13_H_28_O_4_Si2	304.73	0.178 ± 0.035	Malonicacid,bis(2‐trimethylailylethyl)ethyl ester	C_13_H_28_O_4_Si_2_	304.73	0.323 ± 0.064	1‐nonene‐3‐ alcohol	C_9_H_18_O	142.24	0.010 ± 0.001
24	1‐Octane	C_8_H_18_	114.23	0.969 ± 0.323	N‐ Morpholinomethyl ‐isopropyl‐sulfide	C_8_H_17_ONS	175.29	0.432 ± 0.086	1‐nonene‐3‐ alcohol	C_9_H_18_O	142.24	0.009 ± 0.002	N‐ Morpholinomethyl ‐isopropyl‐sulfide	C_8_H_17_ONS	175.29	0.409 ± 0.067
25	2‐hexanone	C_6_H_12_O	100.16	5.932 ± 1.177	1‐ Decene4‐ methyl ‐	C_11_H_22_	154.29	0.034 ± 0.007	N‐ Morpholinomethyl ‐isopropyl‐sulfide	C_8_H_17_ONS	175.29	0.438 ± 0.087	1‐Decene 4‐methyl‐	C_11_H_22_	154.29	0.032 ± 0.007
26	Chloroform	CHC_l3_	119.38	2.723 ± 0.540	Methyl butyrate	C_5_H_10_O_2_	102.13	1.295 ± 0.243	1‐ Decene,4‐methyl‐	C_11_H_22_	154.29	0.034 ± 0.007	Methyl butyrate	C_5_H_10_O_2_	102.13	1.225 ± 0.219
27	Methyl butyrate	C_5_H_10_O_2_	102.13	1.302 ± 0.258	2,4,6‐Trimethyl pyridine	C_8_H_11_N	121.18	0.025 ± 0.005	Methyl butyrate	C_5_H_10_O	102.13	1.312 ± 0.222	2,4,6‐Trimethyl pyridine	C_8_H_11_N	121.18	0.024 ± 0.001
28	1,2‐Benzenedicarboxylic acid bis(2‐methylpropyl) ester;	C_16_H_22_O_4_	278.34	0.215 ± 0.040	Diisooctyladinpate	C_22_H_42_O_4_	370.57	0.064 ± 0.012	2,4,6‐Trimethyl pyridine	C_8_H_11_N	121.18	0.025 ± 0.008	1‐Decene, 3,4‐dimethyl‐	C_12_H_24_	168.32	0.293 ± 0.042
29	Isophthalic acid, dibutyl ester	C_16_H_22_O_4_	278.34	0.090 ± 0.018	Heneicosane‐9‐cyclohexane	C_27_H_54_	378.72	1.105 ± 0.187	1,2‐Benzenedicarboxylic acid bis(2‐methylpropyl)	C_16_H_22_O	278.34	0.217 ± 0.038	1,2‐Benzenedicarboxylic acid bis(2‐methylpropyl) ester;	C_16_H_22_O_4_	278.34	0.202 ± 0.032
30	2‐Hexadecenoic acid	C_16_H_30_O_2_	254.41	0.556 ± 0.108	Tridemorph	C_20_H_33_NO	303.48	0.028 ± 0.009	1‐Decene, 3,4‐dimethyl‐ ester;	C_12_H_24_	168.32	0.313 ± 0.064	Ethyl lactate	C_5_H_10_O_3_	118.13	2.983 ± 0.675
31	Octadecanoic acid	C_18_H_34_O_2_	282.46	0.019 ± 0.003	1‐Decene, 3,4‐dimethyl‐	C_12_H_24_	168.32	0.309 ± 0.06	Diisooctyladinpate	C_22_H_42_O_4_	370.57	0.065 ± 0.012	(Z)‐2‐Octen‐1‐ol	C_8_H_16_O	128.21	0.023 ± 0.005
32	2,5‐Dimethyl furan	C_6_H_8_O	96.13	0.183 ± 0.061	Isopentane	C_5_H_12_	72.15	1.93 ± 0.341	Palmitic acid	C_16_H_32_O_2_	256.42	0.08 ± 0.015	Valeraldehyde	C_5_H_10_O	86.13	0.259 ± 0.053
33	3‐Propyl‐1H‐1,2,4‐ trioazole	C_6_H_8_N_2_O	124.14	0.019 ± 0.004	1,2‐Benzenedicarboxylic acid bis(2‐methylpropyl) ester	C_16_H_22_O_4_	278.34	0.214 ± 0.040	Diisobutyl phthalate 1,2‐Benzenedicarboxylic acid bis(2‐methylpropyl)ester;	C_16_H_22_O	278.34	0.322 ± 0.079	Pentanol	C_5_H_12_O	88.15	0.082 ± 0.017
34	1,1‐dimethyl‐Cyclopentane	C_7_H_14_	98.19	0.089 ± 0.016	Dodecyl aldehyde	C_18_H_34_O_2_	282.46	0.222 ± 0.041	Tridecane	C_13_H_28_	184.36	0.175 ± 0.035	Diisooctyladinpate	C_22_H_42_O_4_	370.57	0.061 ± 0.012
35	3‐Methyl undecane	C_12_H_26_	170.33	0.080 ± 0.015	Eicosane	C_20_H_42_	282.55	0.089 ± 0.022								
36	Tridecane	C_13_H_28_	184.36	0.047 ± 0.009	1,14‐Tetradecanediol	C_14_H_30_O_2_	230.39	0.301 ± 0.060								
37	1,14‐Tetradecanediol	C_14_H_30_O_2_	230.39	0.294 ± 0.073	Cis‐9‐octadecenoate acid	C_18_H_34_O_2_	282.46	2.897 ± 0.513								
38	Cis‐9‐octadecenoate acid	C_18_H_34_O_2_	282.46	2.828 ± 0.561	2‐Hexadecenoic acid	C_16_H_30_O_2_	254.41	0.57 ± 0.131								
39	2,2,3‐trimethyl‐ Hexane,	C_9_H_20_	128.26	2.499 ± 0.443	Octadecanoic acid	C_18_H_34_O_2_	282.46	0.021 ± 0.004								
40	2‐Octenal	C_8_H_14_O	126.20	0.038 ± 0.009	3,5‐Dimethyl furan	C_6_H_8_O	112.12	0.188 ± 0.043								
41	2‐Octene ‐1‐ ol	C_8_H_16_O	128.22	0.036 ± 0.007	3‐Allyl‐1H‐1,2,4‐trioazole	C_6_H_8_N_2_O	124.14	0.02 ± 0.004								
42	Tridecanoic acid	C_13_H_26_O_2_	214.35	0.176 ± 0.041	1,1‐dimethyl‐Cyclopentane,	C_7_H_14_	98.18	0.089 ± 0.020								
43					3‐ Methylundecane	C_12_H_26_	170.33	0.082 ± 0.019								
44					Tridecane	C_13_H_28_	184.36	0.048 ± 0.008								
Sum of relative amount of volatile compounds				30.542				31.966				26.705				25.553

### 3.4 Flavor types of reported volatile compounds in pork meats from different pig breeds

Analysis of volatile compounds in pork meats revealed nine defined classes including hydrocarbons, alcohols, aldehydes, acids, ketones, esters, sulfides, furans, and phenols, as well as small amounts of other compounds such as alkenes and pyrroles. Most compounds were hydrocarbons, followed by acids, esters, aldehydes, and alcohols, and other compounds such as ketones, sulfides, phenols, and furans (Table 5). Flavor compound types that were different in each breed respectively were 42, 44, 34, and 34 in SUS, BAM, YOK, and HZP (Table 5).

### 3.5 Fragrance types of reported flavor compounds in pork meats from different pig breeds

3‐Methyl‐1‐butanol, 1‐nonanal, octanal, hexanal, 2‐pentyl‐furan, 1‐penten‐3‐one, N‐morpholinomethyl‐isopropyl‐sulfide, methyl butyrate, and (E,E)‐2,4‐decadienal were found in all four breeds (Table 5). Chloroform and 2‐hexanone were found only in SUS. 3,5‐Dimethylfuran was found only in BAM. Acetic acid was found in the SUS, BAM, and YOK. Finally, 1‐octen‐3‐ol was found in SUS, YOK, and HZP (Table 5).

## 4 DISCUSSION

There are two main types of compounds responsible for meat flavor: water‐soluble components and lipids (Khan, Jo, & Tariq, 2015). Carbohydrates are derived from the homolytic cleavage of alkoxy radicals in fatty acids. Alcohols are produced from oxidative degradation of fats. Alcohols and carboxylic acids are condensed to form esters, in which oil flavors are dominant (Shahidi, 2001). It was found that lamb flavor was related to heptan‐2‐one and oct‐1‐en‐3‐one amounts, while rancid or undesirable flavors were not related to carbonyl compound abundance (Resconi et al., 2010). Miyasaki et al. assessed several fish samples using an electronic nose system and GC‐MS with HS‐SPME, and showed that some aldehydes and alcohols increased rapidly in jack mackerel and chub mackerel, slowly in skipjack, and slightly in red sea bream and puffer during storage (Miyasaki, Hamaguchi, & Yokoyama, 2011). Likewise, Kaskonienè, Venskutonis, and Ceksteryt (2008) analyzed honey samples of different botanical origins by HS‐SPME/GC‐MS, and identified 93 compounds. Interestingly, using SPME/GC‐MS, it was found that cooked beef meat contained more than 200 volatile compounds, including 36 key odor‐active compounds (Machiels & Istasse, 2003). 3‐Hydroxy‐2‐butanone, 2,3‐butanedione, and 3‐methyl‐1‐butanol were identified as the most representative compounds generated during meat storage (Perez, Rojo, Gonzalez, & de Lorenzo, 2008).

Rongchang pork meat contains volatile compounds such as aldehydes, alcohols, ketones, acids, ethers, esters, hydrocarbons, sulfur‐containing compounds, amines, nitrogen‐containing compounds, furans, oxygen‐containing compounds, and benzene and its derivatives (Liu & Sun, 2010). It was suggested that interactions between these compounds in the muscles produced water‐soluble and volatile flavor compounds, which together formed the unique flavor of Rongchang pork (Liu & Sun, 2010). It was suggested (Du, 2012) that aldehydes and furans, which are compounds found at high amounts, are the main components of pork flavor. Du, (2012) extracted the flavor compound from fermented pork by SPME followed by separation and identification by GC‐MS: 41 flavor compounds were identified, and the main flavor compounds were aldehydes, alcohols, esters, acids, and ketones. In this study, 108 volatile compounds with known molecular formulas were identified in BAM, followed by 106 compounds in SUS. In YOK and HZP, only 98 compounds were identified from pork meats. The relative amounts of total volatile compounds with known molecular formulas accounted for 73.020%, 99.957%, 75.877%, and 76.996% of all detected compounds in SUS, BAM, YOK, and HZP, respectively. These data suggest that different heredity background is an important factor affecting meat flavor.

It was demonstrated that pretreatment temperature constitutes an important factor in the analysis of volatile components of meat from swine by HS‐SPME and GC‐MS. Indeed, Wang et al. (Wang et al., 2008) stated that 50 flavor compounds were detected when samples were pretreated at 60°C and that 168 were found with pretreatment at 80°C. Therefore, 80°C was selected for this study in order to identify the highest number of compounds.

Based on fragrance types of some compounds described in previous reports, the following kinds of fragrance were identified: delicate fragrance (chloroform); apple blossom fragrance: (2‐hexanone); vinegar fragrance (acetic acid); sulfur and fish fragrance (3,5‐dimethylfuran); sweet caramel fragrance (methyl butyrate); and bitter fragrance (1‐octen‐3‐ol) (Zhou, Zhao, Ma, & Huang, 2006). Flavor compounds contained in pork meat from all pig breeds were as follows: 3‐methyl‐1‐butanol (pungent fragrance);1‐nonanal (delicate fragrance); octanal (delicate and fresh fragrance, tender fragrance); hexanal (delicate and grass fragrance, related to the smell of newly mown grass, also having the bad smell of green beans); 2‐pentyl‐furan (ham‐like fragrance); 1‐penten‐3‐one (onion fragrance and barbecue fragrance), (E,E)‐2,4‐decadienal (broth smell) (Chen & Xi); and N‐morpholinomethyl‐isopropyl‐sulfide (important source of meat taste) (Flores, Grimm, Toldr, & Spanier, 1997). Compounds that have a relatively significant contribution for meat flavor are furans, aldehydes, and sulfur‐containing compounds. Indeed, it has been demonstrated that furans are mainly produced from olefinic alcohols and play an important role in the formation of meat flavor (Tan, 2006).

Some previous studies compared the nutritional and flavor compound profiles between different breeds of pork. Quaresma et al. (2011) showed that the fatty acid profile of wild *Sus scrofa scrofa* was similar to that of commercial pork. Sales and Kotrba (2013) reviewed the differences among wild *S. scrofa* and a number of domestic breeds. Of course, *S. scrofa* tended to be smaller than the industrial breeds and to have different meat characteristics. Another study showed the diversity of volatile compounds and that profound differences in these compounds between wild boars and domestic pigs, as shown in the present study (Lammers, Dietze, & Ternes, 2009). These differences could be due to differentially expressed genes. Indeed, it has been shown that 23 genes involved in fatty acid metabolism, intramuscular fat deposition, and skeletal muscle growth were expressed at different levels in SUS compared with YOK (Yang et al., 2016). Nevertheless, additional studies are necessary to determine the contribution of these genes to the volatile compound profile of these breeds.

The present study is not without limitations. Indeed, some components were not identified, indicating that further research is required. In addition, SPME at 80°C might not exactly reflect the conditions at which pork is routinely processed for food. Finally, it would have been helpful to carry out such studies using a range of temperatures for SPME.

In conclusion, the tenderness, taste, succulence, and broth flavor of the BAM and HZP were good. In the SUS, except that succulence was moderate, the other indicators were good. Meanwhile, all the indicators of the YOK pigs were moderate. HS‐SPME/GC‐MS identified many kinds of compounds in longissimus muscle samples from different pig breeds including the crossbreed SUS (106 compounds), and the purebreds BAM (108 compounds), YOK, and HZP (98 compounds each). The volatile compounds in pork belong to several classes, and the highest relative amount of volatile compounds was found in BAM. The main volatile compounds in pork which may contribute to flavor of pork were 3‐methyl‐1‐butanol, 1‐nonanal, octanal, hexanal, 2‐pentyl‐furan, 1‐penten‐3‐one, N‐morpholinomethyl‐isopropyl‐sulfide, methyl butyrate, and (E,E)‐2,4‐decadienal.

## CONFLICT OF INTEREST

The authors declare that they have no conflicts of interest.

## AUTHOR CONTRIBUTIONS

Conception and design of the research: GC; acquisition of data: HW; analysis and interpretation of data: SS; statistical analysis: HW; obtaining funding: GC; drafting the manuscript: LH; revision of manuscript for important intellectual content: YS. All authors read and approved the final manuscript.

## ETHICAL STATEMENT

All studies were performed with the approval of the Animal Use and Care Committee of Gansu Agricultural University, China.

## Supporting information


** **
