# Supplementary figures and images for: Analysis of volatile compounds in pork from four different pig breeds using headspace solid‐phase micro‐extraction/gas chromatography–mass spectrometry
Source: Food Sci Nutr. 2019 Mar 27;7(4):1261–73. doi: 10.1002/fsn3.955 (PMC6475761; doi:10.1002/fsn3.955)

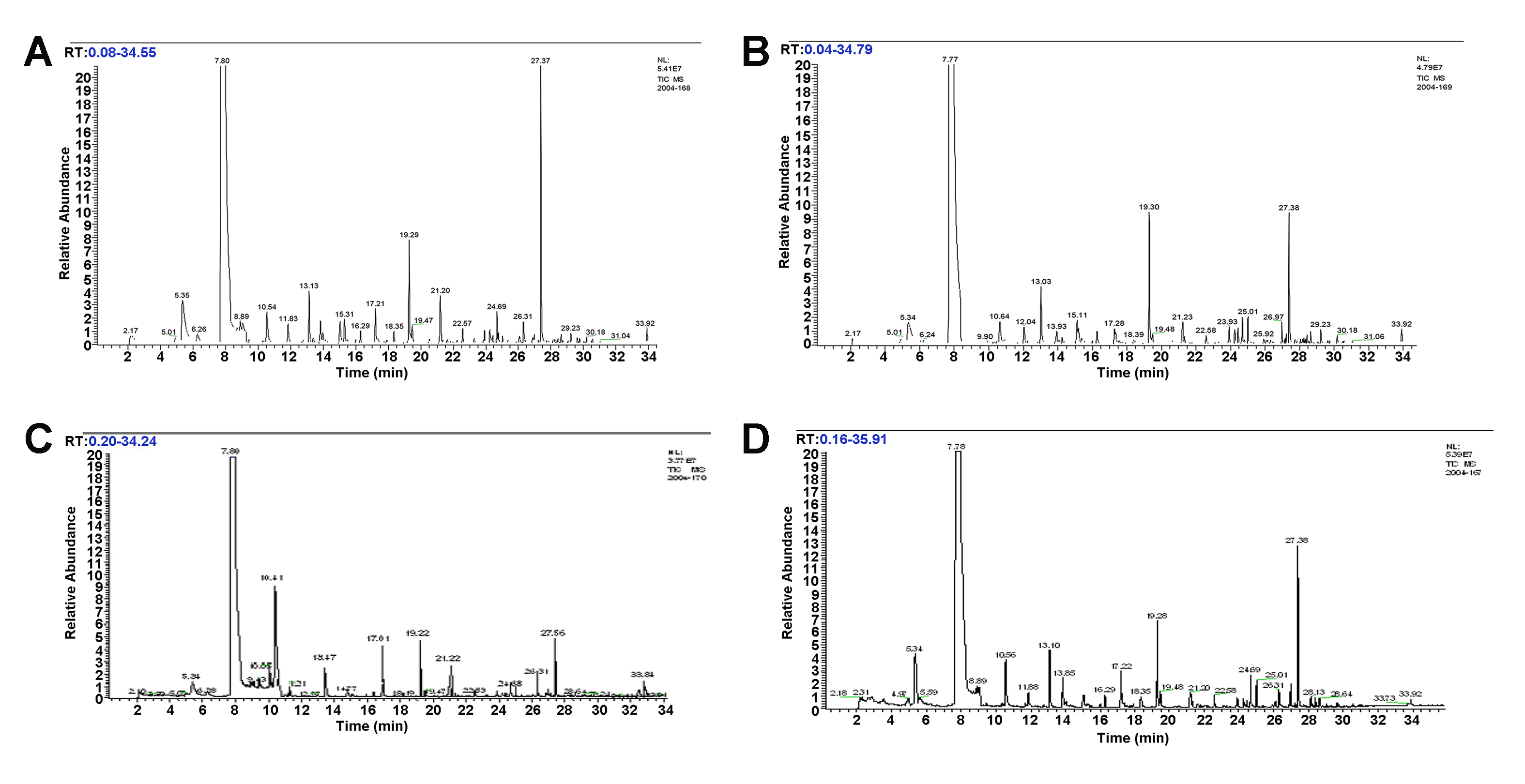

Supplement: Supplementary file 1 [file FSN3-7-1261-s001.tif]
